# Repeatability of dental shade by digital spectrophotometry in current, former, and never smokers

**DOI:** 10.1007/s10266-022-00692-x

**Published:** 2022-03-10

**Authors:** Gianluca Conte, Sebastiano Antonio Pacino, Salvatore Urso, Rosalia Emma, Eugenio Pedullà, Fabio Cibella, Martina Stefanini, Giovanni Zucchelli, Riccardo Polosa

**Affiliations:** 1 Department of General Surgery and Medical-Surgical Specialties, University of Catania, Catania, Italy; 2 Addendo Srl, Dental Clinic, Catania, Italy; 3 ECLAT Srl, Spin-off of the University of Catania, Catania, Italy; 4 Department of Biomedical and Biotechnological Sciences, University of Catania, Catania, Italy; 5 Center of Excellence for the Acceleration of HArm Reduction (CoEHAR), University of Catania, Catania, Italy; 6 Research Council of Italy, Institute of Biomedicine and Molecular Immunology, Palermo, Italy; 7 Department of Biomedical and Neuromotor Sciences, University of Bologna, Bologna, Italy; 8 Department of Clinical and Experimental Medicine, University of Catania, Catania, Italy

**Keywords:** Smoking, Smoking cessation, Dental shade, Digital spectrophotometer, CIE L*a*b*, Whiteness index for dentistry, Reproducibility

## Abstract

Cigarette smoking contributes to poor oral health and dental discoloration. Therefore, stopping smoking may translate into measurable amelioration of dental shade indices. We compared dental shade parameters by digital spectrophotometry among current, former, and never smokers and verified their repeatability at 7 and 30 days. Dental shade parameters (CIE L*a*b* and corresponding whiteness index for dentistry-WID) were measured in current, former, and never smokers with a digital spectrophotometer (Vita Easyshade V) on three separate study visits: at baseline (day 0), at day 7, and day 30. Dental shade parameters were analyzed in 18 current, 18 former, and 20 never smokers. The repeatability of shade parameters was consistent in current, former, and never smokers. L*, a*, b*, and WID show significant short and long-term repeatability (*p* < 0.0001, by regression analyses). The mean (± SD) WID score of 13.42 (± 4.9) in current smokers was significantly lower compared to the WID score of 20.38 (± 5.3) in never smokers (*p* = 0.001). No significant differences were observed between current and former smokers and between former smokers and former smokers. Dental shade measurements by digital spectrophotometry were highly reproducible and showed that teeth whiteness of current smokers is substantially inferior compared to never smokers. Objective discrimination of dental shade can be a valuable regulatory science endpoint for investigating oral hygiene and dental aesthetics of consumer care products, smoking cessation medications, and tar-free tobacco products (e-cigarettes, heated tobacco products, oral nicotine products) for cigarette substitution.

Clinical trial registration: the study was not registered in ClinicalTrials.gov considering that it is a pilot study, parts of a larger project with ID: NCT04649645

## Introduction

Cigarette smoking is known to contribute to poor oral health and tooth discoloration [1, 2]. The particulate matter of cigarette combustion (known as “tar”) contains pigments that can stain and discolor human tissue, including skin, fingernails, and teeth [3–5]. The extent of smoke-related discoloration of the teeth may depend on the intensity and duration of exposure to cigarette smoke [6, 7]. Of note, intrinsic characteristics of the tooth may also contribute; for example, rough/irregular enamel surfaces can facilitate tar adhesion [8].

Teeth color and appearance vary widely [9]; human eye perception, lighting conditions, tooth translucency and opacity, and individual differences in tooth color can contribute to this variability. Therefore, teeth’ visual color determination varies significantly, leading to inaccurate evaluation [10, 11], and tooth shade guides have been introduced to minimize variability. Dentists evaluate teeth color and assess how much whiter teeth can get after using various interventions (e.g., toothbrush, mouthwash or whitening solutions) using tooth shade guides. However, studies with tooth shades guide have shown poor inter-observer correspondence [12, 13] with only 25–35% accuracy for human visual determination of teeth shades [14, 15]. Human shade evaluation cannot be considered for high-quality analysis and research applications, and more precise and reproducible techniques are required.

Innovative technologies for color measurement have been recently introduced to allow more accurate measurements of dental shade. Digital spectrophotometers are considered the most reliable, practical and versatile color matching system [16], with much greater accuracy of dental shade matching than human eye observation [17].

The*a*b* color space principle, developed in 1976 by the Commission International de l’Eclairage (CIE), is used in most studies on tooth color determinations with digital spectrophotometers [18]. L* expresses the intensity of lightness reflected by an object, whereas a* and b* express the defined chromaticity measure of green/red (for a*) and blue/yellow (for b*)–the so-called “forbidden colors” of human vision. The whiteness index for dentistry (WID) has been validated to show that it can accurately capture visual whiteness differences using the CIE L*a*b* color space [19]. Moreover, clinical studies have demonstrated that WID outperformed previous similar indices when assessing the effectiveness of different bleaching procedures on natural teeth [20, 21]. A visually perceivable difference in whiteness between two teeth occurs when WID difference is > 2.90 WID units [22].

To the best of our knowledge, changes in the dental shade in smokers who quit smoking have never been reported. Our research hypothesis is that stopping smoking can translate into measurable amelioration of dental shade indices. However, smoking-induced dental discoloration may be permanent, with limited whiteness restoration after quitting smoking. If this is true, former smokers’ dental shade values should not be much different from current smokers. Moreover, more information is needed to validate within subjects repeatability of digital spectrophotometry in current and former smokers. This is mandatory to increase confidence in the value of this technology for future clinical research.

The objective of the study is to: (a) verify the short (7 days) and long term (30 days) repeatability of dental shade indices obtained by digital spectrophotometry; and (b) compare these measurements between current, former, and never smokers. CIE L*a*b* color space parameters and corresponding WID values were considered for the analyses.

## Methods

### Study population

The study population consists of three study groups identified among a pool of subjects who attended a smoking cessation clinic (CPCT, Centro per la Prevenzione e Cura del Tabagismo of the University of Catania) in the previous 2 years or contacted among hospital staff.

Study group 1 consisted of current smokers, defined as smokers of > 10 cigarettes per day with an exhaled carbon monoxide (eCO) level of ≥ 7 ppm.

Study group 2 consisted of former smokers, defined as quitters of at least 12 months and who were still abstinent when contacted for enrollment, with an eCO level of < 7 ppm.

Study group 3 consisted of never smokers, defined as having never smoked or who reported having smoked less than 100 cigarettes in their lifetime [23]. Their eCO had to be < 7 ppm to exclude subjects passively exposed to cigarette smoke or environmental sources of carbon monoxide.

Current, former, and never smokers had to satisfy the following inclusion criteria:

1. Healthy adult subjects (age 18–50 yrs)
2. Presence of at least ten natural anterior teeth (cuspid to cuspid, lower and upper jaw), with no composite restorations and no prosthetics or crown

Furthermore, they had to satisfy the following exclusion criteria:

1. Any conditions that could interfere with dental shade measurements, including:
  1. Regular daily use of mouth rinse containing essential oil (EO), cetylpyridinium chloride (CPC), or chlorhexidine (CHX) for at least the preceding seven days before screening visit
  2. Subjects wearing fixed or removable orthodontic appliances or prostheses (limited to the 12 natural anterior teeth)
2. Significant exposure to passive smoking (excludes current smokers)
3. Had undergone professional dental cleaning within six months before screening
4. Pregnancy

The study was conducted according to the Principles of Good Clinical Practice (GCP) and Declaration of Helsinki. The local Ethics Committee reviewed and approved the study.

### Study design

**Fig. 1:**
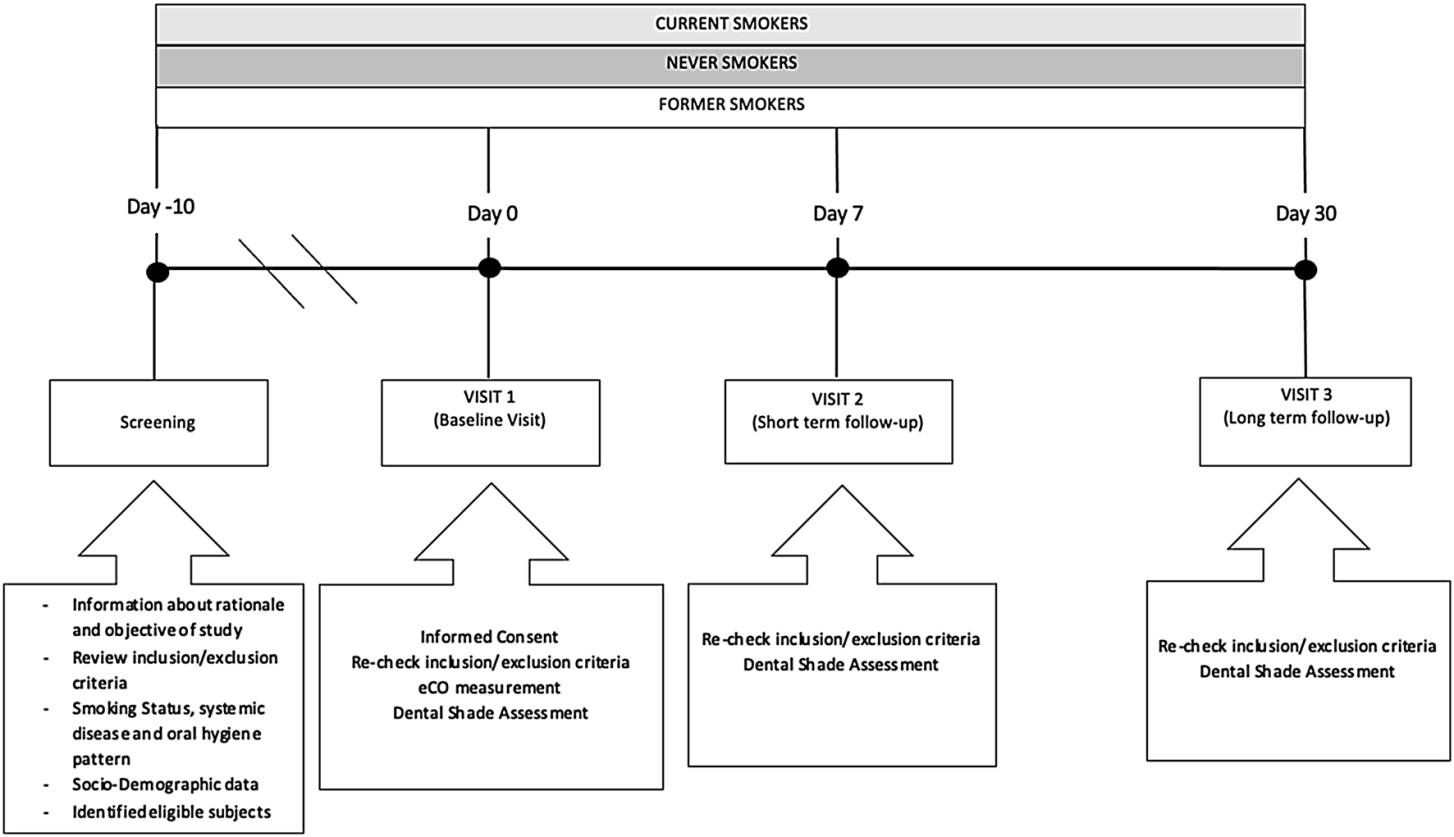
Study Design

This is an observational study to compare digital spectrophotometric parameters for dental shade evaluation by Vita Easyshade V among three study populations (current, former, and never smokers) and to assess their repeatability. The study consists of a total of four visits: screening visit, baseline visit at day 0 (Visit 1), short-term follow-up visit at day 7 (± 1 days) (Visit 2), and a long-term follow-up visit at day 30 (± 3 days) (Visit 3) (Fig. 1). Subjects were asked to:

1. Not to change their habitual oral hygiene (toothbrushing, mouth washing, interdental flossing) pattern for the whole duration of the study
2. Avoid scaling and polishing procedures for the entire period of the study
3. Not to daily use mouth rinse for the whole of the course of the study
4. Not to smoke for at least 2 h before each study visit
5. Not to toothbrush for at least 2 h before each study visit
6. Not to eat and drink (except water) for at least 2 h before each study visit

## Study visits

### Screening visit

Potential participants attended a screening visit to (1) receive information about the rationale and objectives of the research; (2) verify eligibility criteria by reviewing their inclusion and exclusion criteria; (3) assess smoking status and oral hygiene habit (i.e., frequency of toothbrushing, type of toothpaste, etc.); and (4) record general socio-demographic characteristics (i.e., sex, age, and occupation). All eligible subjects were invited to participate in Baseline Visit (Visit 1).

### Baseline visit (visit 1)

Carried out within ten days of the Screening Visit. Subjects were asked to go over a patient information sheet and sign a consent form. After re-checking inclusion/exclusion criteria and reviewing study restrictions, eCO measurement and dental shade assessment were carried out, and baseline data were recorded. Subjects were instructed not to change their habitual oral hygiene pattern and invited to attend the next study visit (Visit 2).

### Day-7 visit (visit 2)

Carried out 7 (± 1) days after Visit 1. Eligibility criteria were verified again. Dental shade assessment was repeated for short-term repeatability. Subjects were instructed not to change their habitual oral hygiene pattern and invited to attend the next study visit (Visit 3).

### Day-30 visit (visit3)

Carried out 30 (± 3) days after Visit 1. After re-checking eligibility criteria, a dental shade assessment was repeated for long-term repeatability.

### Exhaled carbon monoxide measurement

The smoking status was objectively verified by measuring exhaled carbon monoxide (eCO) levels (eCO > 7 ppm indicating smoking status) with a portable CO monitor (Micro CO; Micro Medical Ltd, UK). Subjects were asked not to smoke cigarettes for at least 2 h before eCO measurements. Subjects were invited to exhale slowly into a disposable mouthpiece attached to the eCO monitor per the manufacturer's recommendations. The value of eCO readings was noted.

### Dental shade assessment

Before dental shade assessment, participants were asked to rinse their mouths with water. They were subjected to gentle flushing and drying by triple syringe to remove any food debris.

All measurements were performed in the same examination room, under the exact ambient illumination condition, and by the same operator (GC). The digital spectrophotometer (Vita Easyshade V) was calibrated and used according to the manufacturer’s instructions.

Subjects were instructed to open their mouths with the tongue away from the anterior teeth.

The shade was measured at the central tooth area of the vestibular surface in “base shade determination” mode, with the measuring tip staying at 90° on the tooth surface to achieve an accurate measurement. Subjects were asked to withhold breathing for a few seconds during measurements, to avoid fogging of the measuring tip as this could cause inaccurate shade readings.

CIE L*a*b* color parameters were measured for the vestibular aspect of each anterior tooth (cuspid to cuspid, upper and lower jaw).

The coordinate L* measures lightness, ranging from 0 (black) to 100 (white); the a* and b* coordinates express chromaticity measures of green/red and blue/yellow, respectively. For each subject, total CIE L*a*b* scores were obtained by adding the individual values of each tested anterior tooth and dividing by the number of teeth examined.

### Whiteness index for dentistry (WID)

The whiteness index for dentistry (WID), which is based on CIE L*a*b* coordinates, was calculated as the following equation [18]:

$${\text{WID}}\, = \,0.{\text{511 L}}*{-\!\!-}{2}.{\text{324 a}}*{-\!\!-}{1}.{1}00{\text{ b}}*$$

Each subject’s total WID score was obtained by adding the value of each tested anterior tooth and dividing by the number of teeth examined. High WID values indicate whiter teeth, while low WID values indicate discolored or less white teeth. Differences in the WID index were evaluated in consideration of the whiteness 50:50% acceptability threshold (WAT = 2.90 ΔWID units) [22]; someone can visually appreciate a clear variation in whiteness between two teeth if the ΔWID difference is > 2.90 units.

### Data analysis

Short-term repeatability of CIE L*a*b* and WID scores was evaluated by linear regression analysis of measurements obtained at V1 and at V2 for each study group. Long-term repeatability was assessed by linear regression analysis of measurements at V1 and at V3. Scatter plots of linear regression analyses summarized repeatability results. Moreover, “Bland and Altman” plots were created to illustrate the level of agreement between V1 vs. V2 and V1 vs. V3 for each study group. A 1-tailed sample *t* test was performed to assess the mean difference between two measurements from zero.

Normality was assessed using Shapiro–Wilk’s normality test. Counts and percentages summarized categorical data; continuously distributed data, with symmetrical distribution, were summarized using the mean (standard deviation; SD); continuously distributed data, with skewed distribution, was summarized using the median (interquartile range; IQR). Clinical data comparisons among the groups were carried out by Chi-square test for categorical data and Kruskal–Wallis test for continuously skewed data. Comparison of L* was performed using the Kruskal–Wallis test followed by Wilcoxon pairwise test with Bonferroni’s adjustment for multiple comparisons. Comparisons of a*, b*, and WID scores were performed by one-way ANOVA followed by Tukey's adjustment for multiple comparisons. Moreover, multi-way ANOVA was performed to assess the interaction of age, gender, daily toothbrushing frequency, weekly mouth-washing frequency, and weekly dental flossing frequency on WID score among the study groups.

All analyses were considered significant with a *P* value < 0.05. R version 3.4.3 (2017-11-30) was utilized for data analysis and generation of graphs.

## Results

### Study participants

We screened a total of 70 subjects: five failed eligibility screening and four failed to attend their baseline visit. Therefore, 61 subjects were enrolled in this study, of which 5 participants were excluded from statistical analysis after failing to attend their short- or long-term follow-up visit.

Complete analysis of dental shade assessment was carried out in 56 subjects (21 F; mean ± SD age of 34.73 ± 11.5 years) 18 current, 18 former, and 20 never smokers (Table 1).

**Table 1:** Clinical characteristic of study groups

	Current smokers	Former smokers	Never smokers	*p* value
Subjects n	18	18	20	
Age	31 (26–37)	32 (25–43)	34 (29.5–37)	0.871
Female	4/17 (23.5%)	7/17 (41.2%)	10/19 (52.6%)	0.202
Exhaled CO	15 (11–14.9)	3 (3–4)	3 (2–4)	< 0.0001
n. Cigarette/day	15 (10–15)	//	//	NA
Pack/years	9.6	4.7	//	NA
Year non-smoking	//	2 (1.5–9)	//	NA
Toothbrushing frequency/daily	2 (1.5–3)	2 (2–3)	2 (2–2.25)	0.454
Mouth washing frequency/daily	0 (0–0)	0 (0–0)	0 (0–2)	0.008
Dental flossing frequency/weekly	0 (0–1.5)	1 (0–1.5)	1 (0–1.5)	0.791

Data are presented as median (IQR), n/N (%)

*CO* carbon monoxide

### Dental shade repeatability in never smokers

Linear regression analyses were performed to assess short term (7 days) and long term (30 days) repeatability of dental shade parameters for never smokers. Analyses are summarized in Table 2.

**Table 2:** Dental shade parameters repeatability analysis in Never Smokers

Parameters	7 days (short term) repeatability		30 days (long term) repeatability	
	Regression analysis V2-V1 R value (*p* value)	Mean of the difference V2-V1 being different from zero? YES/NO (*p* value)	Regression analysis V3-V1 R-value (*p* value)	Mean of the difference V3-V1 being different from zero? YES/NO (*p* value)
L*	0.9287 (*p* = 0.0001)	NO (*p* = 0.080)	0.8673 (*p* = 0.0001)	NO (*p* = 0.169)
a*	0.9146 (*p* = 0.0001)	NO (*p* = 0.890)	0.8644 (*p* = 0.0001)	NO (*p* = 0.443)
b*	0.8690 (*p* = 0.0001)	NO (*p* = 0.233)	0.8633 (*p* = 0.0001)	YES (*p* = 0.004)
WID	0.9293 (*p* = 0.0001)	NO (*p* = 0.884)	0.8672 (*p* = 0.0001)	NO (*p* = 0.338)

### Short-term repeatability

For L*, a significant regression was observed between study visits (R^2^ = 0.9287; *p* = 0.0001). No subjects had the L* difference between V1 and V2 outside the 95% confidence interval, and the mean of differences between L* at V2 and V1 was not different from zero (p = 0.080). Also, for the parameters a* and b* we observed significant regression between study visits (R^2^ = 0.9146, 0.0001 for a*; R^2^ = 0.8690, *p* = 0.0001 for b*). Only two subjects had the * difference between V1 and V2 outside the 95% confidence interval. Moreover, the mean differences between a* at V2 and V1 were not different from zero (*p* = 0.890). Only one subject had the b* difference between V1 and V2 outside the 95% confidence interval. Moreover, the mean differences between b* at V2 and V1 were not different from zero (*p* = 0.233).

**Fig. 2:**
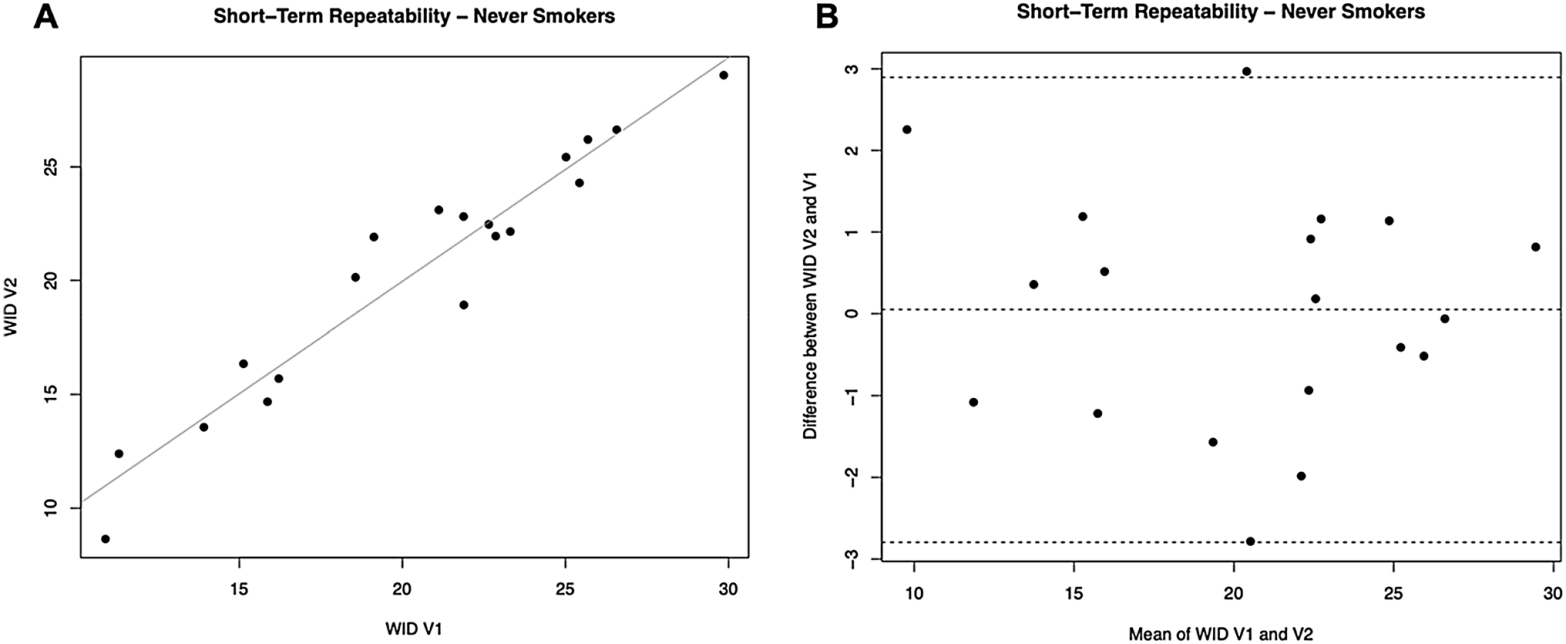
Short-term repeatability (V2 vs. V1) of WID scores in Never Smokers. **A** shows the scatter plot of regression analysis between visit 2 (V2) and visit 1 (V1) for WID scores. **B** shows the difference between the measurements taken at V1 and V2 concerning the mean in each subject in the Bland Altman plot

For WID scores, a significant regression was observed between study visits (R^2^ = 0.9293; *p* = 0.0001) (Table 2, Fig. 2A). Only one subject had the WID difference between V1 and V2 outside the 95% confidence interval (Fig. 2B). Moreover, the mean differences between WID at V2 and V1 were not different from zero (*p* = 0.884).

### Long-term repeatability

For L*, a significant regression was observed between study visits (R^2^ = 0.8673; *p* = 0.0001). Only one subject had the L* difference between V1 and V3 outside the 95% confidence interval. The mean differences between L* at V3 and V1 were not different from zero (*p* = 0.169). Also, for the parameters a* and b* we observed significant regression between study visits (R^2^ = 0.8644, *p* = 0.0001 for a*; R^2^ = 0.8633, *p* = 0.0001 for b*). Only one subject had the * difference between V1 and V3 outside the 95% confidence interval. Moreover, the mean differences between a* at V3 and V1 were not different from zero (*p* = 0.443). Only one subject had the b* difference between V1 and V3 outside the 95% confidence interval. However, the mean differences between b* at V3and V1 were significantly different from zero (*p* = 0.004).

**Fig. 3:**
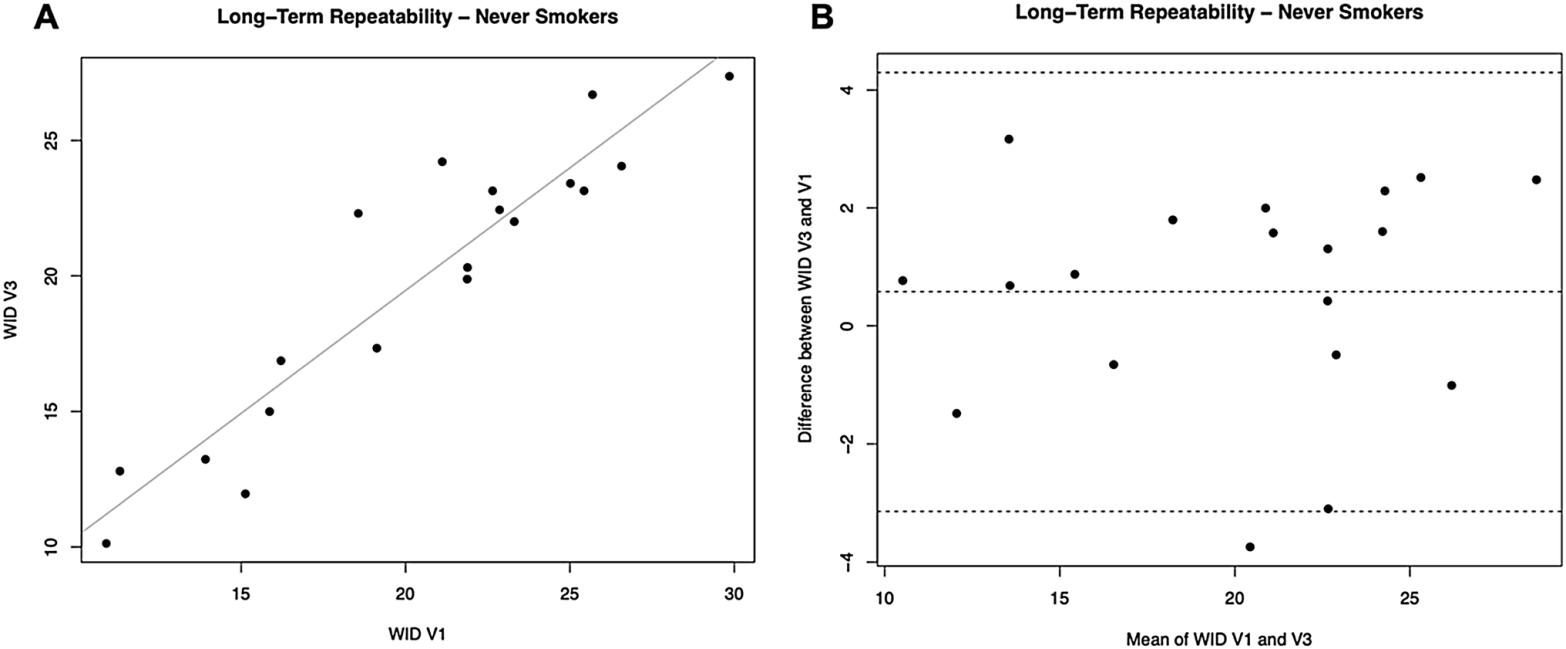
Long-term repeatability (V3 vs. V1) of WID scores in Never Smokers. **A** shows the scatter plot of regression analysis between visit 3 (V3) and visit 1 (V1) for WID scores. **B** shows the difference between the measurements taken at V1 and V3 concerning the mean in each subject in the Bland Altman plot

For WID scores, a significant regression was observed between study visits (R^2^ = 0.8672; *p* = 0.0001) (Table 2, Fig. 3A). Only one subject had the WID difference between V1 and V3 outside the 95% confidence interval (Fig. 3B). Moreover, the mean differences between WID at V3 and V1 were not different from zero (*p* = 0.338).

## Dental Shade Repeatability in Current Smokers

Linear regression analyses were performed to assess short term (7 days) and long term (30 days) repeatability of dental shade parameters for current smokers. Analyses are summarized in Table 3.

**Table 3:** Dental shade parameters repeatability analysis in Current Smokers

Parameters	7 days (short term) repeatability		30 days (long term) repeatability	
	Regression analysis V2-V1 R value (*p* value)	Mean of the difference V2-V1 being different from zero? YES/NO (*p* value)	Regression analysis V3-V1 R-value (*p* value)	Mean of the difference V3-V1 being different from zero? YES/NO (*p* value)
L*	0.9495 (*p* = 0.0001)	NO (*p* = 1)	0.9038 (*p* = 0.0001)	NO (*p* = 0.712)
a*	0.9603 (*p* = 0.0001)	NO (*p* = 0.6)	0.9455 (*p* = 0.0001)	NO (*p* = 0.394)
b*	0.8573 (*p* = 0.0001)	NO (*p* = 0.446)	0.8033 (*p* = 0.0001)	YES (*p* = 0.012)
WID	0.9076 (*p* = 0.0001)	NO (*p* = 0.773)	0.8292 (*p* = 0.0001)	NO (*p* = 0.4)

### Short-term repeatability

For L*, a significant regression was observed between study visits (R^2^ = 0.9495; *p* = 0.0001). Only one subject had the L* difference between V1 and V2 outside the 95% confidence interval, and the mean of differences between L* at V2 and V1 was not different from zero (*p* = 0.110). Also, for the parameters a* and b* we observed significant regression between study visits (R^2^ = 0.9603, 0.0001 for a*; R^2^ = 0.8573, *p* = 0.0001 for b*). One subject had the * difference between V1 and V2 outside the 95% confidence interval. Moreover, the mean differences between a* at V2 and V1 were not different from zero (*p* = 0.660). One subject had the b* difference between V1 and V2 outside the 95% confidence interval. Moreover, the mean differences between b* at V2 and V1 were not different from zero (*p* = 0.446).

**Fig. 4:**
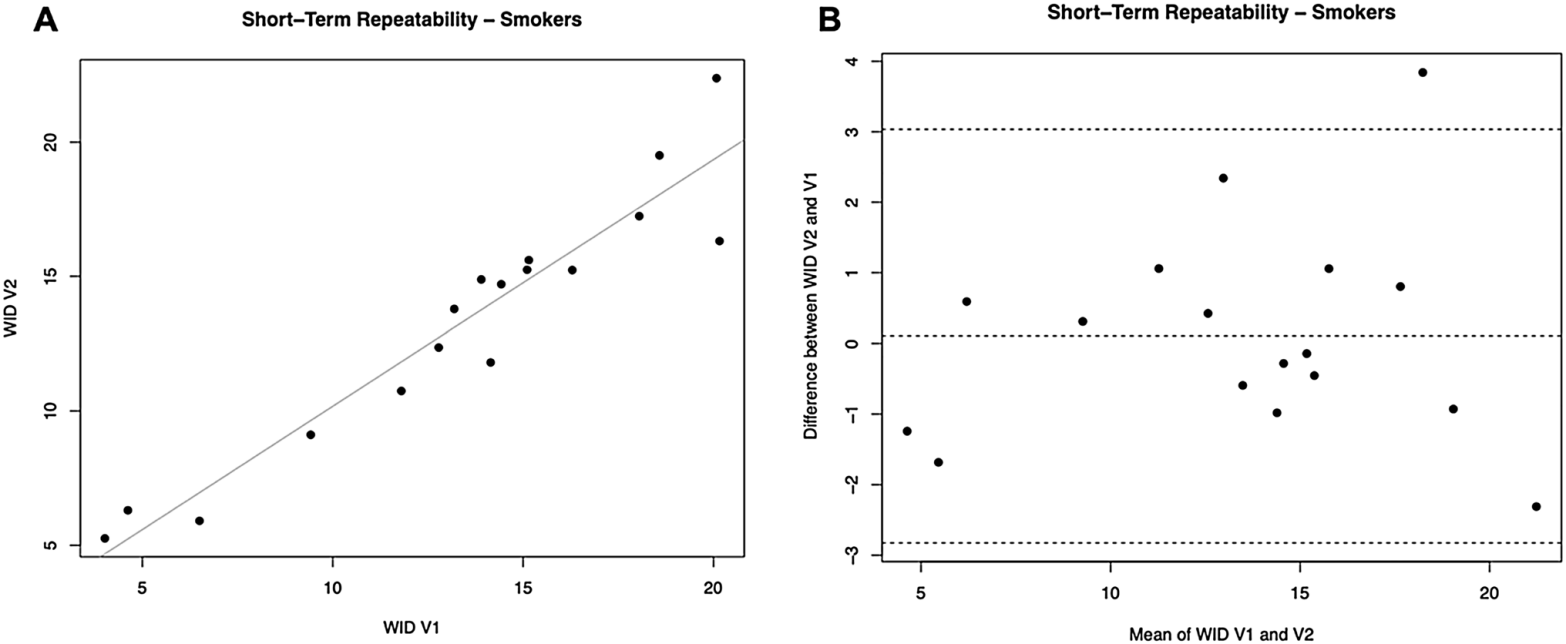
Short-term repeatability (V2 vs. V1) of WID scores in Current Smokers. **A** shows the scatter plot of regression analysis between visit 2 (V2) and visit 1 (V1) for WID scores. **B** shows the difference between the measurements taken at V1 and V2 concerning the mean in each subject in the Bland Altman plot

For WID scores, a significant regression was observed between study visits (R^2^ = 0.9076; *p* = 0.0001) (Table 3, Fig. 4A). Only one subject had the WID difference between V1 and V2 outside the 95% confidence interval (Fig. 4B). Moreover, the mean differences between WID at V2 and V1 were not different from zero (*p* = 0.773).

### Long-term repeatability

For L*, a significant regression was observed between study visits (R^2^ = 0.9038; *p* = 0.0001).

No subjects had the L* difference between V1 and V3 outside the 95% confidence interval. Moreover, L* the mean differences between L* at V3 and V1 were not different from zero (*p* = 0.712). Also, for the parameters a* and b* we observed significant regression between study visits (R^2^ = 0.9455, *p* = 0.0001 for a*; R^2^ = 0.8033, *p* = 0.0001 for b*). One subject had the * difference between V1 and V3 outside the 95% confidence interval. Moreover, the mean differences between a* at V3 and V1 were not different from zero (*p* = 0.394). Two subjects had the b* difference between V1 and V3 outside the 95% confidence interval, and the mean of the differences between b* at V3 and V1 was significantly different from zero (*p* = 0.012).

**Fig. 5:**
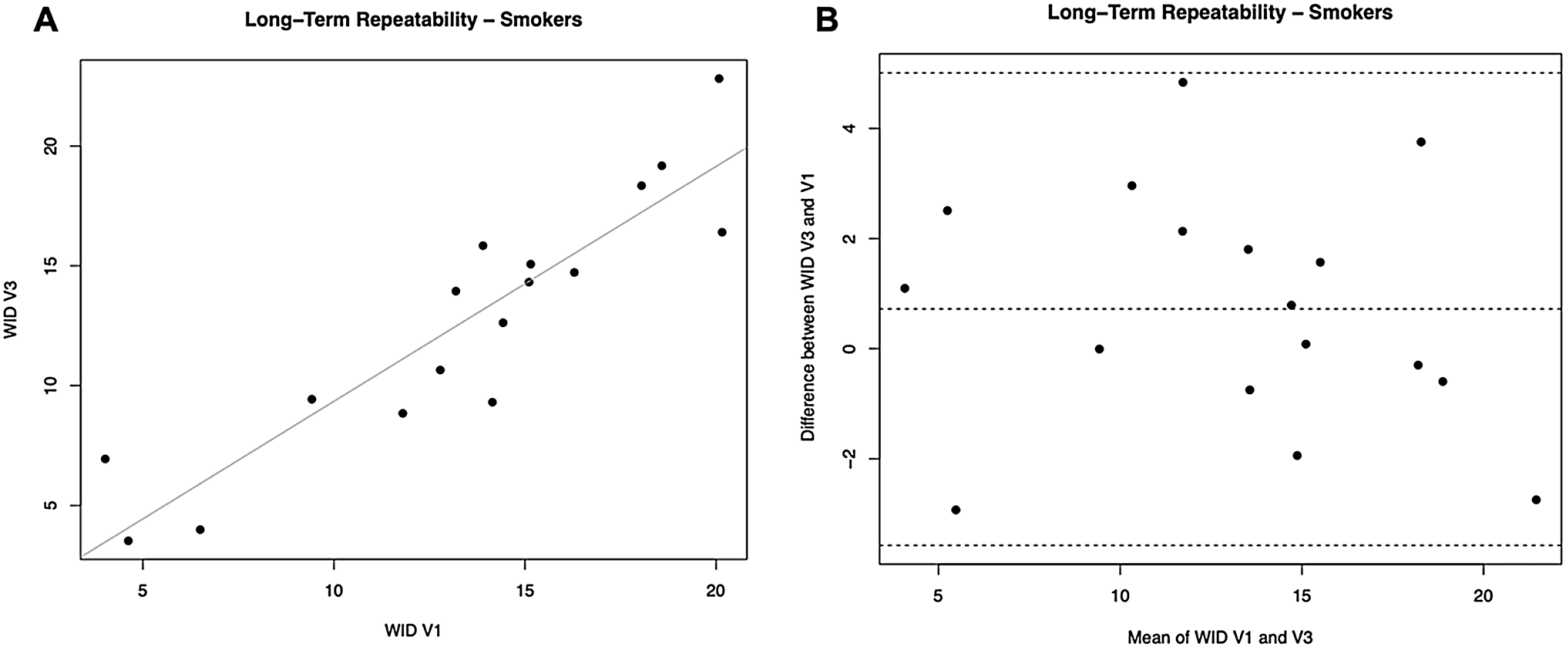
Long-term repeatability (V3 vs. V1) of WID scores in Current Smokers. **A** shows the scatter plot of regression analysis between visit 3 (V3) and visit 1 (V1) for WID scores. **B** shows the difference between the measurements taken at V1 and V3 concerning the mean in each subject in the Bland Altman plot

For WID scores, a significant regression was observed between study visits (R^2^ = 0.8292; p = 0.0001) (Table 3, Fig. 5A). No subjects had the WID difference between V1 and V3 outside the 95% confidence interval (Fig. 5B). Moreover, the mean differences between WID at V3 and V1 was not different from zero (p = 0.424).

## Dental shade repeatability in former smokers

Linear regression analyses were performed to assess short- and long-term repeatability of dental shade parameters for former smokers. Analyses are summarized in Table 4.

**Table 4:** Dental shade parameters repeatability analysis in Former Smokers

Parameters	7 days (short term) repeatability		30 days (long term) repeatability	
	Regression analysis V2-V1 R value (*p* value)	Mean of the difference V2-V1 being different from zero? YES/NO (*p* value)	Regression analysis V3-V1 R-value (*p* value)	Mean of the difference V3-V1 being different from zero? YES/NO (*p* value)
L*	0.9256 (*p* = 0.0001)	NO (*p* = 0.927)	0.9215 (*p* = 0.0001)	NO (*p* = 0.548)
a*	0.9846 (*p* = 0.0001)	NO (*p* = 0.278)	0.9692 (*p* = 0.0001)	NO (*p* = 0.921)
b*	0.9646 (*p* = 0.0001)	YES (*p* = 0.012)	0.9103 (*p* = 0.0001)	NO (*p* = 0.102)
WID	0.9855 (*p* = 0.0001)	YES (*p* = 0.018)	0.9492 (*p* = 0.0001)	NO (*p* = 0.408)

### Short-term repeatability

For L*, a significant regression was observed between study visits (R^2^ = 0.9256; *p* = 0.0001).

No subjects had the L* difference between V1 and V2 outside the 95% confidence interval, and the mean of differences between L* at V2 and V1 was not different from zero (*p* = 0.927). Also for the parameters a* and b* we observed significant regression between study visits (R^2^ = 0.9846, 0.0001 for a*; R^2^ = 0.9646, *p* = 0.0001 for b*). No subjects had the * difference between V1 and V2 outside the 95% confidence interval, and the mean of differences between a* at V2 and V1 was not different from zero (*p* = 0.278). Only one subject had the b* difference between V1 and V2 outside the 95% confidence interval. However, the mean differences between b* at V2 and V1 were significantly different from zero (*p* = 0.012).

**Fig. 6:**
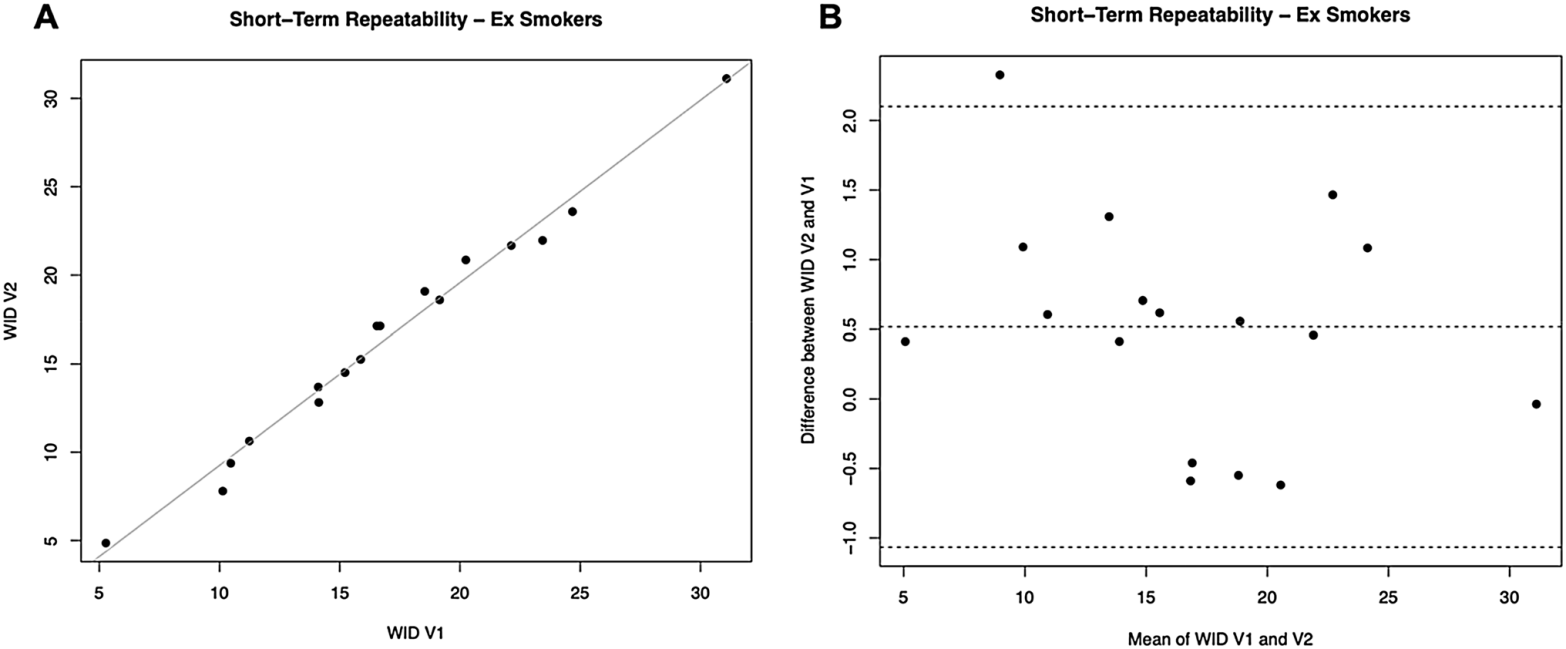
Short-term repeatability (V2 vs. V1) of WID scores in Former Smokers. **A** shows the scatter plot of regression analysis between visit 2 (V2) and visit 1 (V1) for WID scores. **B** shows the difference between the measurements taken at V1 and V2 concerning the mean in each subject in the Bland Altman plot

For WID scores, a significant regression was observed between study visits (R^2^ = 0.9855; *p* = 0.0001) (Table 4, Fig. 6A). Only one subject had the WID difference between V1 and V2 outside the 95% confidence interval (Fig. 6B). However, L* the mean differences between L* at V2 and V1 were significantly different from zero (*p* = 0.038).

### Long-term repeatability

For L*, a significant regression was observed between study visits (R^2^ = 0.9215; *p* = 0.0001).

No subjects had the L* difference between V1 and V3 outside the 95% confidence interval. Moreover, the mean differences between L* at V3 and V1 were not different from zero (*p* = 0.548). Also, for the parameters a* and b* we observed significant regression between study visits (R^2^ = 0.9692, *p* = 0.0001 for a*; R^2^ = 0.9103, *p* = 0.0001 for b*).

Only one subject had the * difference between V1 and V3 outside the 95% confidence interval. Moreover, the mean differences between a* at V3 and V1 were not different from zero (*p* = 0.921). No subjects had the b* difference between V1 and V3 outside the 95% confidence interval, and the mean of differences between b* at V3 and V1 was not different from zero (*p* = 0.102).

**Fig. 7:**
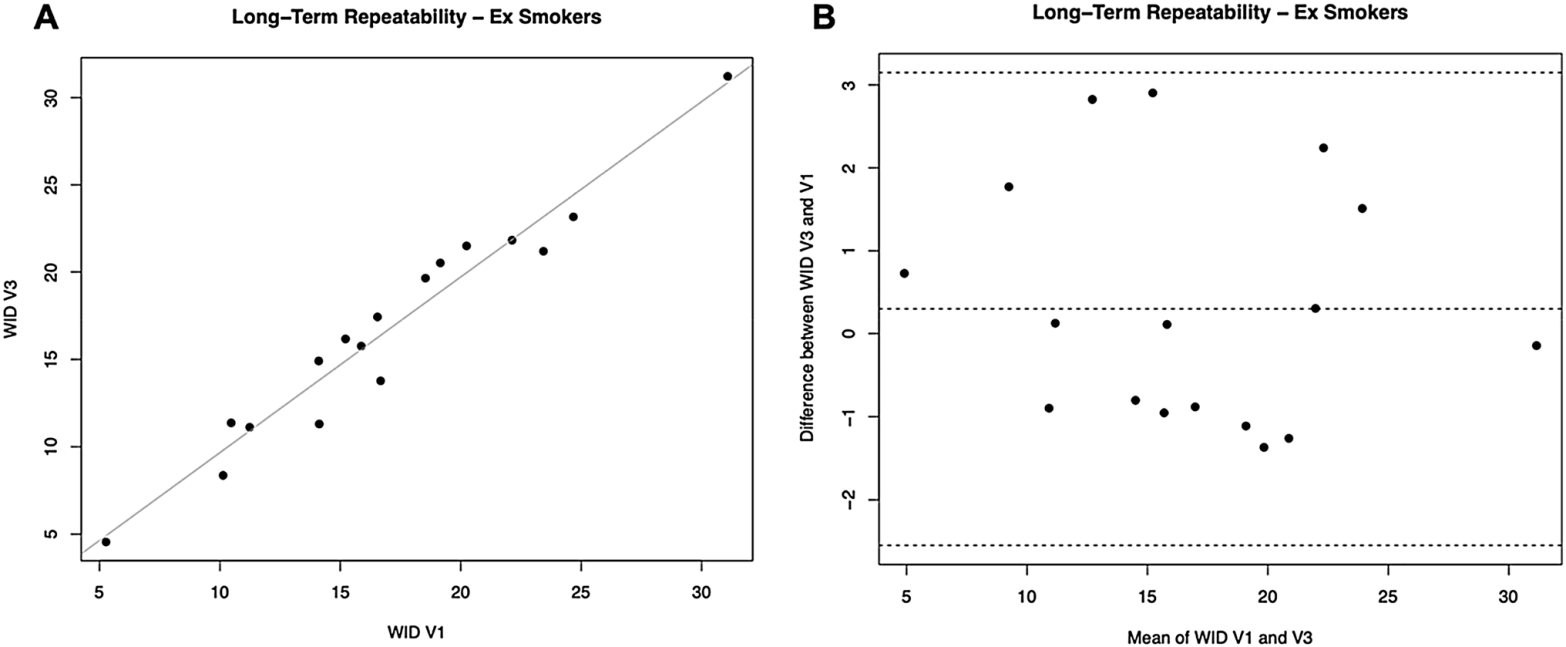
Long-term repeatability (V3 vs. V1) of WID scores in Former Smokers. **A** shows the scatter plot of regression analysis between visit 3 (V3) and visit 1 (V1) for WID scores. **B** shows the difference between the measurements taken at V1 and V3 concerning the mean in each subject in the Bland Altman plot

For WID scores, a significant regression was observed between study visits (R^2^ = 0.9492; *p* = 0.0001) (Table 4, Fig. 7A). No subjects had the WID difference between V1 and V3 outside the 95% confidence interval (Fig. 7B). Moreover, the mean differences between WID at V3 and V1 were not different from zero (*p* = 0.408).

## Summary of repeatability analysis

In all study groups (current, former, and never smokers), dental shade parameters L*, a*, b*, and WID showed significant short- and long-term repeatability. Validation of the repeatability of digital spectrophotometry can increase confidence in the value of this technology for future clinical research.

## Comparison between current, former, and never smokers

Median (IQR) and Mean (± SD) values of L*, a*, b*, and WID scores for the three study groups are summarized in Table 5. The L*values were similar, and no significant difference was observed among the three study groups (*p* = 0.475). Moreover, no significant difference in the L* score was observed when cross-comparisons were performed (*p* values > 0.05). This means that tooth lightness (L*) seems not to be affected by smoking habits. Significant differences were observed between study groups for both a* (*p* = 0.016) and b* (*p* < 0.001), meaning that smoking cigarettes could alter tooth color, increasing yellowness (b*) and redness (a*) perception and causing tooth discoloration.

**Table 5:** Comparison of L*, a*, b*, and WID scores among Current, Former, and Never smokers

	L*	a*	b*	WID
	Median (IQR)	Mean ± SD	Mean ± SD	Mean ± SD
Current smokers	79.69 (73.9–81.5)	0.57 ± 0.8	22.71 ± 2.8	13.42 ± 4.9
Former smokers	79.78 (77.9–83.3)	0.26 ± 0.99	20.87 ± 2.9	16.99 ± 6.2
Never smokers	80.15 (77.6–83.1)	− 0.22 ± 0.6	18.92 ± 3	20.38 ± 5.3
Overall *P* value	*P* = 0.475*	*P* = 0.02^§^	*P* = 0.001^§^	*P* = 0.002^§^

Data are presented as median (interquartile range) or mean ± standard deviation (SD). The overall *p* values were calculated by Kruskal–Wallis Test* or one-way ANOVA^§^

**Fig. 8:**
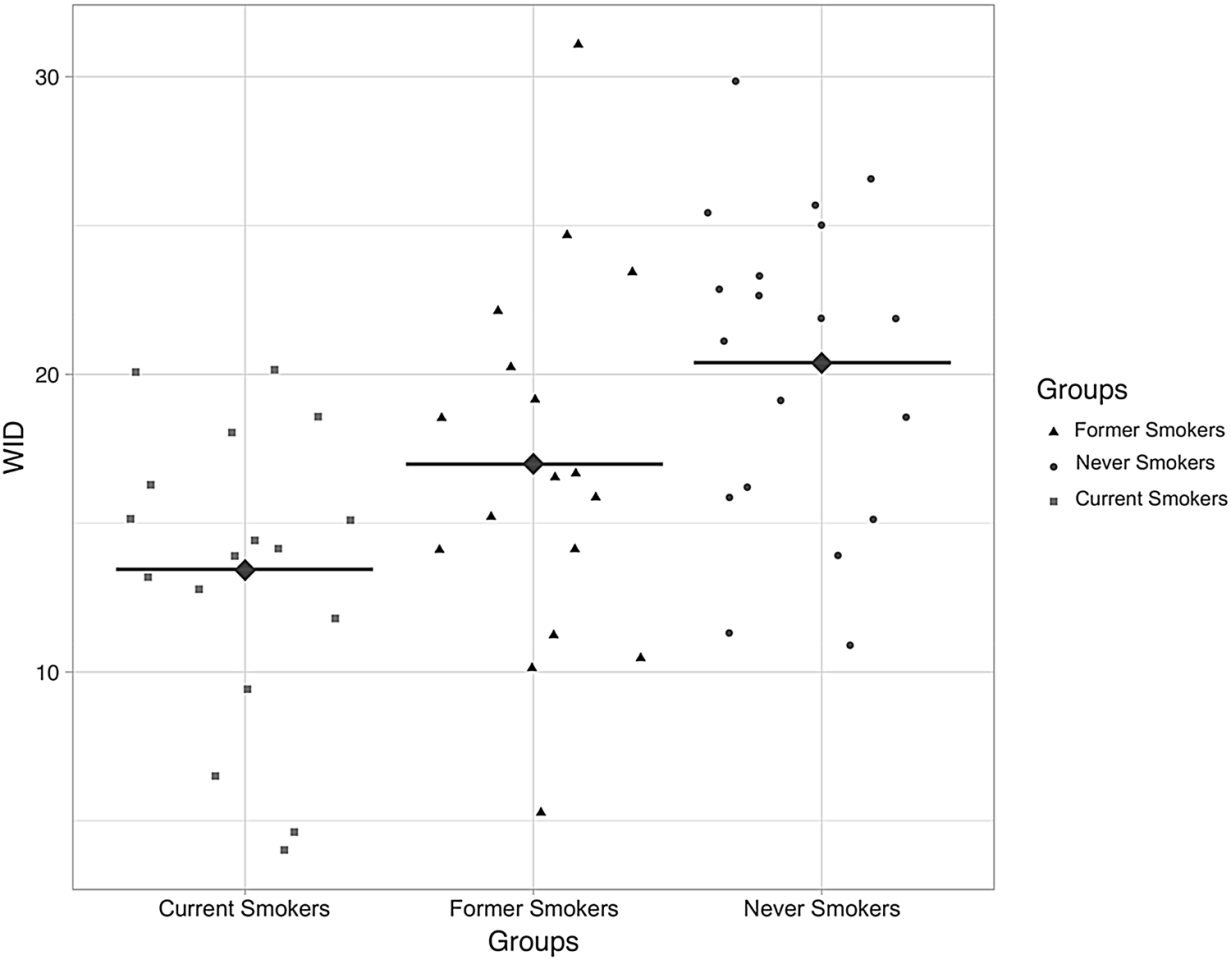
Individual WID scores among Current, Former, and Never smokers. The diamond points with a black horizontal bar illustrate the means of each study group. High WID values indicate whiter teeth, with low WID values showing discolored or much darker teeth

A significant difference was observed for WID scores among the study groups (*p* = 0.002) (Fig. 8). The mean (± SD) WID score of 13.42 (± 4.9) in current smokers was significantly lower compared to the WID score of 20.38 (± 5.3) in never smokers (*p* = 0.001), indicating discolored or much darker teeth. No significant differences in WID scores were observed between current smokers and former smokers (*p* = 0.153) and between never smokers and former smokers (*p* = 0.167).

## Assessment of interaction effects on WID score

A statistically significant simple main effect of age for WID score (p = 0.042). No other meaningful interactions were observed for gender, daily toothbrushing frequency, weekly mouth-washing frequency, and weekly dental flossing frequency on WID score among the study groups.

## Discussion

Avoiding cigarette smoke toxicants may translate into measurable amelioration of teeth appearance. Information about changes in dental shade indexes in smokers who quit smoking is not available. This study is the first to investigate the impact of smoking and smoking abstinence on teeth appearance by comparing digital spectrophotometry measurements of dental shade in current, former, and never smokers. Dental shade measurements showed that the teeth whiteness of current smokers is substantially inferior compared to never smokers.

Former and current smokers had lower WID values (indicating discolored or less white teeth), with a statistically significant difference being observed only between current and never smokers, but not between current and former smokers. Therefore, dental discolorations caused by smoking may be persistent, with only negligible improvement after stopping smoking. An alternative explanation for the lack of significant difference between current and former smokers is that the relatively short (2 years on average) duration of smoking abstinence in our sample of former smokers was not long enough to allow a complete appreciable reversal of dental discoloration. Moreover, it is possible that the observed trend could have become statistically significant with a larger sample size.

The reported difference of teeth whiteness between current smokers and never smokers were not only statistically significant but also of practical/clinical relevance because it largely exceeded the 2.90 ΔWID units’ threshold, above which a visual difference in whiteness between two teeth starts to be apparent [22]. The observation that teeth whiteness of current smokers is substantially inferior compared to never smokers was not unexpected; the conclusion is consistent with what we know about pigments in the particulate matter of cigarette smoke and its staining of human tissues [3–5]. Our clinical findings are compatible with recent experimental work with human premolars extracted for orthodontic reasons [6] and bovine enamel blocks [7], showing that cigarette smoke induces substantial dental discoloration.

A few factors and limitations need to be considered when interpreting these study findings. First, the study populations consisted of relatively young subjects, and their dental shade measurements may not be representative of the general population. This is particularly important, considering that age was the only factor causing a statistically significant interaction with WID scores. Consequently, additional studies with more representative age groups are needed to confirm our findings. Second, dental shade measurements were performed only on the vestibular aspect of each anterior tooth (cuspid to cuspid, upper and lower jaw). However, it is unlikely that study findings would have changed significantly by extending measurements to all existing natural teeth (including the lingual/palatal portion). Third, COVID-19 restrictions had only a minimal impact on the study conduct because it was carried out between the first and second wave of the pandemic in Italy (July 2020–October 2020) when clear dental settings guidelines were already set in place and most restrictions to hospital access were lifted.

Last but not least, this is a proof-of-concept pilot study, which has been conducted to validate the repeatability of a new methodology and increase researchers’ confidence in the value of this measurement for clinical research, including our ongoing large multicentre randomized controlled trial [24]. No previous data for WID values could be used for power calculation. However, based on experimental studies (6, 7) the extent of smoke-related discoloration of the teeth is so considerable that a small sample size can be adequate. This has been confirmed by the data of the current study; when comparing dental shade parameters of 18 current smokers with those of 20 never smokers, mean WID scores resulted significantly different (13.42 for current smokers vs. 20.38 for never smokers; *p* = 0.001). Therefore, a sample of 18–20 subjects is adequate to detect significant and aesthetically relevant differences (WID difference was well above 2.9 units—the visually perceivable whiteness difference between two teeth). However, it is possible that the absence of significant differences with former smokers (i.e., current smokers vs. former smokers; never smokers vs. former smokers) could be due to a small sample size and that a much larger sample could have detected significant differences. When considering within-subject repeatability, the narrow confidence intervals indicate very low measurement variability suggesting that a small group of 18–20 subjects can be informative.

This study also confirms the good reproducibility of dental shade indices measured by VITA Easyshade V, both short term and long term; our findings are in agreement with those obtained with the same digital spectrophotometer in several studies [25–27].

This is the first study to investigate dental shade repeatability in current, former, and never smokers. Study findings show significant short- and long-term repeatability in all study groups for all dental shade indices (except L*). In particular, a high level of repeatability was found for WID score with significant regression between study visits (both short and long term) in all study groups.

The issue of test variability is significant when investigating dental outcomes in clinical trials of subjects with significant exposure to tobacco smoke. We believe that the good reproducibility of dental shade measurements in this study was due to (1) optimization of environmental conditions with standardization of ambient light; (2) careful consideration of factors that could significantly affect study measurements (e.g., asking participants not to alter the habitual pattern of their oral hygiene practices, avoiding fogging of the measuring tip during measurements); (3) well-trained operators performing the test correctly and accurately, and (4) using an accurate and reliable digital spectrophotometer.

Dental shade measurements by digital spectrophotometry were highly reproducible and showed that teeth whiteness of current smokers is substantially inferior compared to never smokers. These findings may have important implications for smoking cessation, especially for those smokers for which bad breath and teeth appearance (due to tooth discoloration and “tar”/tobacco stains) are often perceived as a significant problem [28–30]. Moreover, objective, reproducible discrimination of dental shade measurements will increase confidence in their value for a range of applications, including clinical and regulatory research applied to combustion-free tobacco products (e.g., e-cigarettes, heated tobacco products, oral tobacco/nicotine products), smoking cessation medications, and consumer care product for oral hygiene and dental aesthetics.
